# *Hymenopleella* and *Diaporthe* dominate the fungal community of dieback affected sea Buckthorn from Northern Germany

**DOI:** 10.1186/s40793-025-00804-4

**Published:** 2025-11-14

**Authors:** Carolin Popp, Falk H. Behrens, Alicia Balbín-Suárez, Michael Fischer, Wilhelm Jelkmann, Sabine Kind

**Affiliations:** 1 Julius Kuehn-Institute - Federal Research Centre for Cultivated Plants Institute for Plant Protection in Fruit Crops and Viticulture, Schwabenheimer Str. 101, Dossenheim, Germany; 2 Julius Kuehn-Institute - Federal Research Centre for Cultivated Plants Institute for Plant Protection in Fruit Crops and Viticulture, Geilweilerhof, Siebeldingen, Germany

**Keywords:** Hippophae rhamnoides, Canker, Hymenopleella, Diaporthe, Random forest modelling

## Abstract

**Background:**

Since recent years, German sea buckthorn (SBT) cultivation is increasingly affected by dieback. Wildly growing plants from dunes and cultivated plants from plantations show symptoms of wilt, lesions and discolorations in shoot cross sections. The cause of final plant death is not yet resolved and asymptomatic plants are rare to find. Our aim was to investigate the associated fungal communities of visibly dieback affected plants. A culture-dependent isolation approach in parallel with a culture-independent sequencing approach by metabarcoding of ITS1 was used to investigate SBT shoot fungal communities. Evaluation of the sequencing data was supported with random forest modelling.

**Results:**

Results of both approaches complement each other and are consistent. Members of the ascomycete genera *Hymenopleella* and *Diaporthe* were most frequently isolated from symptomatic samples. *Alternaria*, *Aureobasidium*, *Cladosporium*,* Epicoccum* and *Penicillium* could be identified in both sample types, i.e. symptomatic and asymptomatic plants, with high frequencies. Sequencing of shoot samples revealed that the fungal community composition differs significantly between symptomatic and asymptomatic plants. Pielou’s evenness was significantly reduced for symptomatic plants indicating a dominance of few fungal taxa in symptomatic samples pointing to a dysbiosis in fungal communities. In a random forest modelling approach, abundance of *Capnocheirides* amplicon sequence variants had the highest relative importance for the model and high relative abundance is considered as predictor for absence of SBT symptoms. In symptomatic plants, *Hymenopleella* and *Diaporthe* had high relative abundances and were suggested as predictors.

**Conclusions:**

Overall, our combined approach has revealed an increased abundance of *Hymenopleella* and *Diaporthe* in symptomatic sea buckthorn in Germany along with changes in the total fungal community. The relative abundances derived from amplicon sequencing were reflected by the isolation frequencies of the respective taxa.

**Supplementary Information:**

The online version contains supplementary material available at 10.1186/s40793-025-00804-4.

## Background

Sea buckthorn (SBT), *Hippophae rhamnoides* L., is a versatile shrub whose berries are processed in food, medicine and cosmetics, containing various valuable components, like vitamins, carotenoids and omega fatty acids with antioxidant and antimicrobial properties [1–3]. In Germany, SBT was also used for soil stabilization in former brown coal mines. Plants from natural sources were selected and planted for coastal protection along the Baltic Sea. A breeding program for yield and pollinator varieties was initiated in the 1970s with ‘Leikora’ as first licensed variety [4, 5]. In 2021, the area of German SBT cultivation amounted to 596 ha [6].

**Fig. 1:**
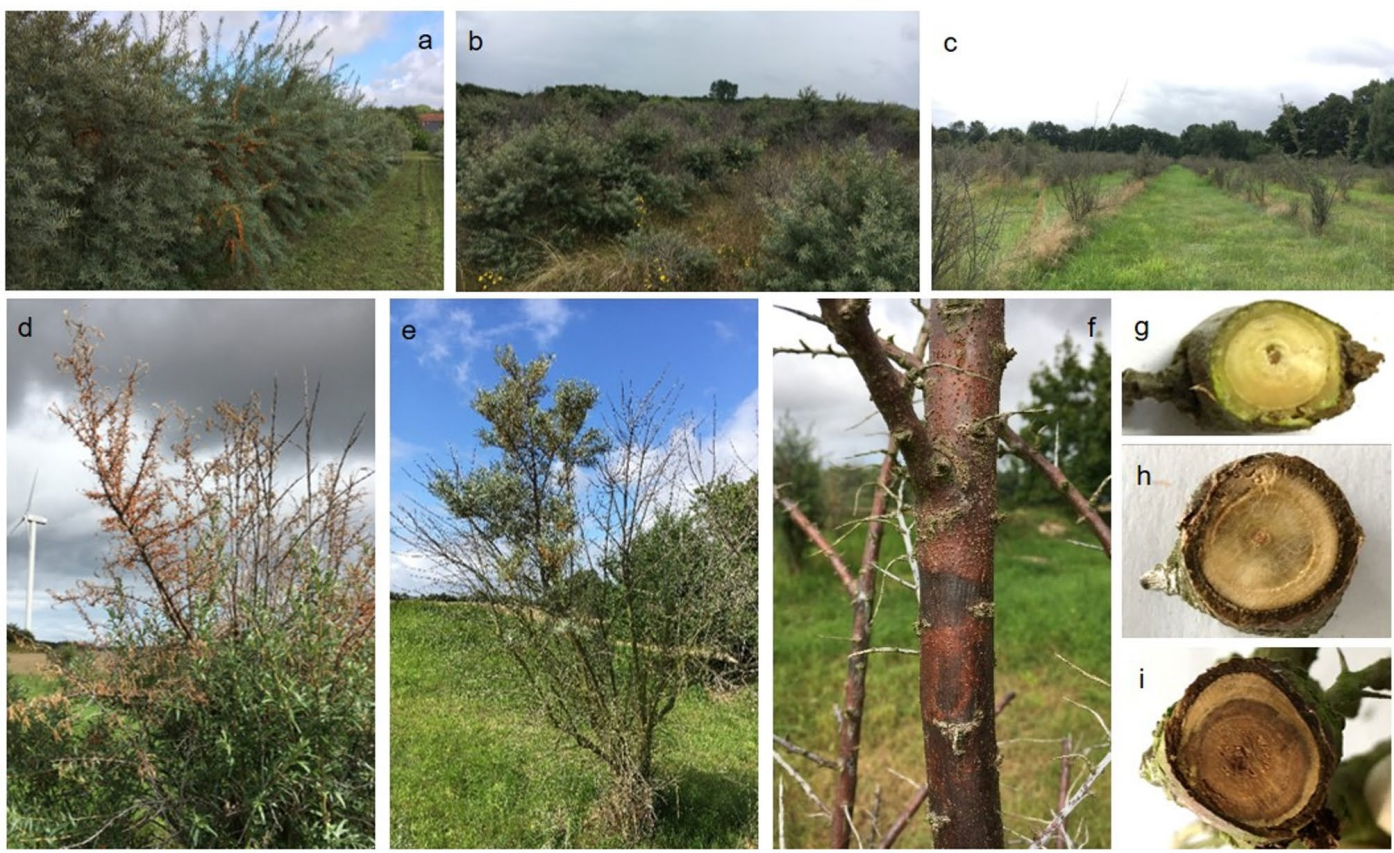
Symptoms of sea buckthorn dieback in Northern Germany. **a** Vital sea buckthorn plants in Gülzow test facility, August 2021; **b** Sea buckthorn plants on a dune in Graal-Müritz (Baltic Sea), August 2021; **c** dieback affected plantation, August 2021; **d** wilt of branches after leave and fruit set in a plantation, August 2021; **e** flagging: plants with vital and dead sections; **f** bark lesions; shoot cross sections **g** asymptomatic cross section, **h** and **i** cross sections with discolorations

Since 2015, an increase of SBT dieback was noted in the North-Eastern part of Germany [7, 8]. Affected are wildly growing plants in dunes and cultivated plants in plantations [9, 10]. SBT dieback had been reported already some decades ago: Darmer [11] described a “typical, extensive death of sea buckthorn plants” along the German Baltic Sea coast, the exact causes of which were questionable, with i.e. impermeable humus layer, change in the soil properties, wind impact, increasing soil acidity, stagnant seawater, caterpillar feeding, root rot, or pathogenic taxa within the fungal family of *Valsaceae* are discussed as possible triggers. Over recent years, the incidence of dieback further increased up to complete losses, resulting in major ecological and economic impact. Due to the extreme spread of dieback already at the beginning of our study in November 2020, asymptomatic plants were almost impossible to find at many sites (Fig. 1a−c). The most typical external symptom is wilt that starts around June and may affect single branches (“flagging”) or the whole plant. Dried out fruits and leaves remain on the shrunken branches (Fig. 1d, e). For some plants, lesions and canker can be observed on the bark surface characterized by brown-reddish color and dead phloem tissue underneath with sunken margins forming a line of separation to the neighboring green tissue (Fig. 1f). Sometimes leaf yellowing can be observed as well. In shoot cross-sections grey to dark brown discolorations can be found as internal symptoms (Fig. 1g-i). All these symptoms are thought to be due to a biological background, however, previous attempts to identify definite causal agents so far failed [12].

SBT has been cultivated for some decades only [11]. Compared to other temperate climate fruit crops that are in cultivation for more than a century like apple, research on SBT diseases is very limited [13]. Drevinska and Moročko-Bičevska [14] reviewed possible fungal pathogens for SBT and reported on similar symptoms as described above for other SBT growing regions, although with less horrendous losses as in Northern Germany. In most cases, the symptoms have been considered caused by complex disorders [14]. Wilt of SBT is widely distributed and is most frequently caused by *Verticillium dahliae* and to a lesser degree by *V. albo-atrum*, e.g. in Belarus, Latvia, Romania, Scotland and Canada [13, 15–18]. In a previous work, *Verticillium* was discussed as main causal dieback agent also in Germany [19]. Additionally, different *Fusarium* species are reported to cause wilt and dieback of SBT, e.g. in Mongolia, India, and Belarus [18, 20–23]. In China, *F*. *sporotrichioides* in the Gansu region [24] and *F*. *proliferatum* in the Liaoning region were proven to cause stem wilt and dying of SBT [25]. The so called dried-shrink disease in China overlaps with symptoms described for wilt and was reported previously [14, 26]. Large areas of Chinese SBT cultivation are affected by dried-shrink disease leading to a reduction of fruit yields and may result in death of the whole shrub. A complex of *F*. *acuminatum*,* F. oxysporum*,* F. camptoceras* and *Diaporthe* spp. (as syn. *Phomopsis*) was reported to cause the dried-shrink disease in China [26, 27]. In addition, *Dothidea hippophaes* (as syn. *Plowrightia hippophaes*) was identified as another potential causal agent in China [14, 28]. In a recent study, Zalewska et al. [29] investigated fungi inhabiting aboveground SBT organs derived from organic plantations in Poland. For these, wilt and leaf yellowing symptoms were described, in this way similar to the plants examined in our study. *Alternaria alternata* and *A*. *radicina* were most frequently isolated and were considered as hazardous, since they were detected at all sampling time points and amounted to 46% and 5.4% of the total isolated fungi.

Another symptom related with SBT dieback is stem canker, associated with several fungal pathogens: Petrak [30] found *Hymenopleella hippophaeicola* (as syn. *Leptosphaeria hippophaës*, further syn. *Lepteutypa hippophaës*) on dry SBT branches in Austria. In Finland, stem canker appeared frequently around buds in different Russian cultivars and was reported to be potentially caused by *Stigmina* sp [31]. *Cytospora* canker has been described for SBT in China and Canada [32, 33]. In Latvia members of *Diaporthe*, *Eutypa*, *Fusarium* and *Verticillium* were associated with canker and decline [13].

Considerable efforts have been made to elucidate the causal agent of SBT dieback, but no particular causal agents could be identified confirming the complex nature of the dieback disease. In diagnostic testing and preliminary work upon the occurrence of the SBT symptoms in Northern Germany, material from symptomatic tissue was also tested for bacteria including phytoplasms and viruses. The preliminary results gave no reason to deepen those investigations within the scope of this study, which is therefore focused on fungi as causal agents of SBT dieback.

The previous studies as mentioned above are based mainly on culture-dependent isolation from affected tissue [13, 15, 26]. In our study a fungal-isolation approach was for the first time combined with a culture-independent metabarcoding approach to investigate fungal communities in SBT. Both approaches were applied to compensate each other for their inherent limitations regarding community completeness, as technical and methodological biases are difficult to eliminate entirely. Amplicon sequencing may overlook certain taxa due to primer mismatches or other constraints in amplicon preparation, while fungal isolation misses obligate biotrophs and underrepresents slow-growing organisms or those with specific culture requirements.

Random forest modelling was applied to test if ASVs and location are among explanatory variables that define the symptomatic state of plant (symptomatic vs. asymptomatic). It enables the inclusion of unknown biological relationships that emerge from statistical patterns in the data. The output of the random forest modelling is the mean relative importance (MRI) for each variable: it indicates how strongly it helps to classify symptomatic vs. asymptomatic samples - calculated from how much each contributes to improving model accuracy on average across all decision trees.

We hypothesized that the communities are significantly altered by SBT dieback and our aim therefore was to compare fungal communities from both affected habitats (environment and plantations) and to identify associated fungal taxa in symptomatic plants.

## Methods

Plant material: A total of 104 sea buckthorn (SBT) plants were sampled (Additional file 1). Of these, 82 showed external shoot symptoms such as wilt, dead branches, bark lesions, leaf yellowing and/or internal symptoms, e.g. wood discolorations in shoot cross sections (Fig. 1). As plants of both habitats (environment and plantation) were already massively dieback-affected at the start of our investigations, asymptomatic plants were rare to find. The number of asymptomatic samples therefore is much lower with overall 22 samples taken from asymptomatic and vital plants, preferably located close to symptomatic plants. From seven plants both symptomatic and asymptomatic parts were sampled independently (overview in Table 1). From each plant or each section, two to three branches taken from different positions were pooled into one sample. Each sample, with the outer bark removed, consisted of a mixture of wood and phloem tissue. If symptoms were present, symptomatic tissue was sampled with adjacent tissue. Sampling was performed from November 2020 until the end of August 2021 with all sampled habitats located in the northern parts of Germany. Shoot samples were taken from wildly growing plants of eleven different environmental locations and cultivated plants from nine plantations. Samples were classified for specific symptoms: (i) bark lesions, (ii) shrinkage and (iii) wood discolorations. Details of the investigated plant material are summarized in Additional file 1.

**Table 1:** Overview of plants from which both tissue types, symptomatic and asymptomatic were sampled

No.	Origin	Plant/sample name	Metabarcoding identifier	
			Symptomatic	Asymptomatic
1	Plantation A, Brandenburg, cv. Leikora	HRS_1_with + without	Meta_002	Meta_001
2		HRS_2_with + without	Meta_004	Meta_003
3		HRS_3_with + without	Meta_006	Meta_005
4		HRS_5_with + without	Meta_008	Meta_007
5	Environment, Ruegen, MV, WP	HRS_150 + 151	Meta_120	Meta_119
6		HRS_153 + 154	Meta_123	Meta_122
7	Environment, Priwall, Schleswig Holstein, WP	HRS_156-2 + 156-3	Meta_127	Meta_126

These samples were used for isolation and metabarcoding approach. MV: Mecklenburg-Western Pomerania; WP: wild plants (unknown cultivar)

Fungal isolation and identification: 71 plants were used for the isolation approach, 59 had visible symptoms and 12 were classified as asymptomatic (Table 2, additional file 2). From seven symptomatic plants, asymptomatic sections were sampled as described above (Table 1). Shoot material (length 4–5 cm) was surface sterilized by short flaming, cut into 0.5 –1 cm pieces and then placed on 2% malt extract agar containing 25 µg ml^-1^ chloramphenicol. From each plant a minimum of four shoot pieces was incubated resulting in 385 pieces from symptomatic and 78 pieces from asymptomatic plants, as well as 34 pieces from asymptomatic sections of symptomatic plants. The Petri dishes were incubated for two weeks at 20 °C in darkness. Macroscopically different mycelia from each sample piece were transferred separately. The total number of isolates amounted to 408, 328 isolates from symptomatic plants, 65 isolates from asymptomatic plants and 15 isolates from asymptomatic sections of symptomatic plants. 185 isolates were identified by molecular means, the remaining 223 isolates were assigned to genus level by macroscopic characters such as color, growth, and overall morphological appearance. The molecular identification of pure cultures was carried out as follows: Approximately 1 cm^2^ of the mycelium was harvested in 2 ml-reaction tubes, shock frozen with liquid nitrogen and stored at -20 °C until further processing. Mycelium was ground in the reaction tube in liquid nitrogen with a small pestle. DNA extraction was carried out by using a silica-based method as described in Menzel et al. [34] followed by ITS-PCR. The 10 µl reaction mix consisted of 5 µl Phusion Flash High Fidelity PCR Master Mix (Thermo Fisher, USA), 1 µl DNA extract and 1 µl of each primer (10 µM), ITS1 and ITS4 [35]. Cycling conditions (C1000 touch thermal cycler, Bio-Rad, USA) were as follows: 98 °C for 10 s, followed by 32 cycles at 98 °C for 1 s, 57 °C for 5 s and 72 °C for 20 s, and final elongation at 72 °C for 5 min. After visualization by gel electrophoresis, the fragments were purified with Monarch PCR & DNA Cleanup Kit (New England Biolabs, USA). Samples were sent for Sanger sequencing to Microsynth Seqlab (Germany). Sequences were evaluated using BLASTn, core nucleotide database (last access: November 2024), megablast (highly similar sequences), choosing 1st hit with maximum score (NCBI, USA). Isolation rate was calculated by dividing the total number of obtained isolates through the number of incubated shoot pieces, comparing symptomatic and asymptomatic samples. Further data evaluation was done qualitatively by only counting one isolate of a genus per plant regardless of the total number of obtained isolates per genus (Additional file 2), counted isolates are marked in bold letters. These data were used to calculate the relative identification of fungal genera by dividing it by the number of plants analyzed in regard of symptoms. Sanger sequences of representative isolates were deposited in the NCBI gene bank (Accession numbers: PP210697 - PP210858, Additional file 3). For *Hymenopleella*, *Diaporthe* and *Fusarium* additional multi-locus PCRs were conducted to enable identification on species level (part of *translation elongation factor 1-alpha* (*Hymenopleella* and *Fusarium* primer EF-1 and EF-2 [36], *Diaporthe* EF1-688 F and EF1-1251R [37] and *β-tubulin* (*Diaporthe* and *Hymenopleella* primer T1 and Bt2b [38, 39].

**Table 2:** Numbers of sea Buckthorn plants

	Isolation approach		Sequencing approach	
	Environment	Plantation	Environment	Plantation
Locations	8	7	11	9
symptomatic	24	35	36	43
asymptomatic	7	5	7	7
Σ	31	40	43	50
Total Σ	71		93	

The total number of sampled plants was 104, 82 symptomatic and 22 asymptomatic (the majority of plants has been used for both approaches - details are given in Additional file 2)

Amplicon sequencing: For amplicon sequencing 93 plants were used (79 symptomatic, 14 asymptomatic). Surface sterilized plant material was ground with a mortar and pestle in liquid nitrogen of which approximately 50 mg were used for DNA extraction with silica particles [34]. DNA extracts were diluted 1:10. DNA metabarcoding library preparation and sequencing were carried out by AllGenetics & Biology SL (www.allgenetics.eu) as in [40]. Illumina sequencing primers were attached at 5’ends to the amplicon primer combination ITS1catta/ITS2ngs [41, 42], which preferably amplify fungal and oomycetes ITS1 region. PCR mix with a total volume of 12.5 µl contained 1.5 µl template DNA, 0.5 µM of each primer, 3.13 µl Supreme NZYTaq 2x Green Master Mix (NZYTech, Portugal) and ultra pure water up to 12.5 µl was incubated as follows: 95 °C for 5 min, followed by 35 cycles at 95 °C for 30 s, 45 °C for 45 s, 72 °C for 45 s, and final elongation at 72 °C for 7 min. In a second amplification step oligonucleotide indices were attached with identical conditions but with an annealing temperature of 60 °C for 5 cycles. Library size was verified by gel electrophoresis, library purification was done by using Mag-Bind RXNPure Plus magnetic beads (Omega Biotek, USA). Libraries were quantified with a Qubit dsDNA HS Assay (Thermo Fisher Scientific, USA), pooled in equimolar amounts according to the obtained data and sequenced in a MiSeq PE300 run (Illumina, USA). Template free library preparations (BPCR) and a negative control of the DNA extraction were included to check for contamination. Amplicon sequencing raw data was submitted to NCBI data base with BioProject and SRA data ID PRJNA1069732 and BioSamples Accession numbers SAMN39620747 - SAMN39621060.

Metabarcoding data processing and analyses: Metabarcoding data processing was carried out as described in [40]. For all metabarcoding data operations R v4.2.1 was used [43]. Packages utilized were *tidyverse* v1.3.2 [44], *devtools* v2.4.5 [45], *Rcpp* v1.0.9 [46], and *usethis* v2.1.6 [47]. After only using forward reads [48], primers were clipped with Cutadapt v3.7 [49] and *ShortRead* v1.54.0 [50]. The *dada2* package v1.24.0 [51] was applied for quality filtering, denoising with independent sample inference, removing of chimeras and taxonomic assignment of amplicon sequence variants (ASVs) against UNITE database v9.0 [52].

Data analyses were carried out with *phyloseq* v1.40.0 [53]. Potential contaminations were excluded by *decontam* v1.16.0 [54]. Additional packages applied were *cowplot* v1.1.1 [55], *dplyr* v1.0.9 [56], *ggplot2* v3.3.6 [57], *ggpubr* v0.4.0 [58], *ggrepel* v 0.9.3 [59], *ggthemes* v4.2.4 [60], gplots v3.1.3 [61], *gridExtra* v2.3 [62], *MicEco* v0.9.19 [63], *multcompView* v0.1-9 [64], *plyr* v1.8.7 [65], *readxl* v1.4.0 [66], *vegan* v2.6-2 [67], *viridis* v0.6.2 [68], and *writexl* v1.4.2 [69]. For analyses, only ASVs of taxa Fungi and Stramenopila were taken into account and individual ASVs had to be present in at least two samples. According to [70], samples with low counts (< 1000 fungal and stramenopila reads) were removed from the analysis, resulting in a set of 93 evaluated plants (Table 2). Of these, 79 samples were symptomatic (36 environment, 43 plantation) and 14 asymptomatic (7 environment, 7 plantation).

For alpha diversity, observed richness (number of different ASVs) and the Pielou’s eveness index (equally abundance of different ASVs) were studied. ANOVA followed by a post-hoc Tukey´s HSD correction test (*P* < 0.05) was calculated to find statistical differences in regards of symptoms (symptomatic vs. asymptomatic), origin (Environment vs. Plantation), location, cultivar and specific symptoms.

For beta diversity analysis, the filtering of ASVs was more strict: ASVs assigned to the same genus were combined and only those present in at least seven samples with min. 10 reads were considered resulting in 73 ASVs. Samples showed a variation in read depth and were rarefied to an even depth of 1000 reads with 100 replications (Additional file 4). Rarefied data were subjected to a Principal Coordinate Analysis (PCoA, Bray-Curtis dissimilarity index) and PERmutational Multivariate ANalysis Of VAriance (PERMANOVA; 10,000 permutations). Beta diversity analysis was conducted to test the influence of the investigated factors (symptom, origin, cultivar and location) on the fungal community composition. Beta diversity of specific symptoms was tested separately.

Rarefied reads were submitted to random forest modelling following the description in [71] using ranger v0.16.0 [72]. ASVs were the same as for beta diversity. These data in combination with the factors origin, location and cultivar were used as explanatory variables to classify the symptomatic status of plants. The best performing hyperparameter combination was calculated in a grid search (number of trees: 1-750; Mtry: number of features *c (0.1, 0.15, 0.2, 0.25, 0.333, 0.4); minimum node size: 1,3,5; sample fraction: 0.7, 0.8, 0.9; feature importance measure “impurity”) and evaluated by comparing random forest out-of-bag (OOB) errors.

As the number of plant samples were unbalanced for the factor symptom, stratified sampling was performed to split the data in training and test sets (proportion = 0.9).

The mean relative importance of each ASV and factor for the prediction of the symptomatic status was calculated from 100 random forest models, all based on the best hyperparameter combination and differing training sets. For the variables with the highest mean relative importance (≥ 1%), relative abundance and log_2_ fold change were calculated. For *Hymenopleella*, *Diaporthe* and *Capnocheirides* the relative abundance is displayed in boxplots comparing symptoms and origin of samples.

## Results

Differences between fungal communities of dieback affected and asymptomatic sea buckthorn plants should be identified by the use of fungal isolations and ITS-based metabarcoding sequencing. After taxon identification the communities were compared regarding isolation rate, alpha and beta diversity, correlation with symptoms and by random forest modelling, factors were ascribed relative importance as explanatory variables for the presence or absence of symptoms.

**Fig. 2:**
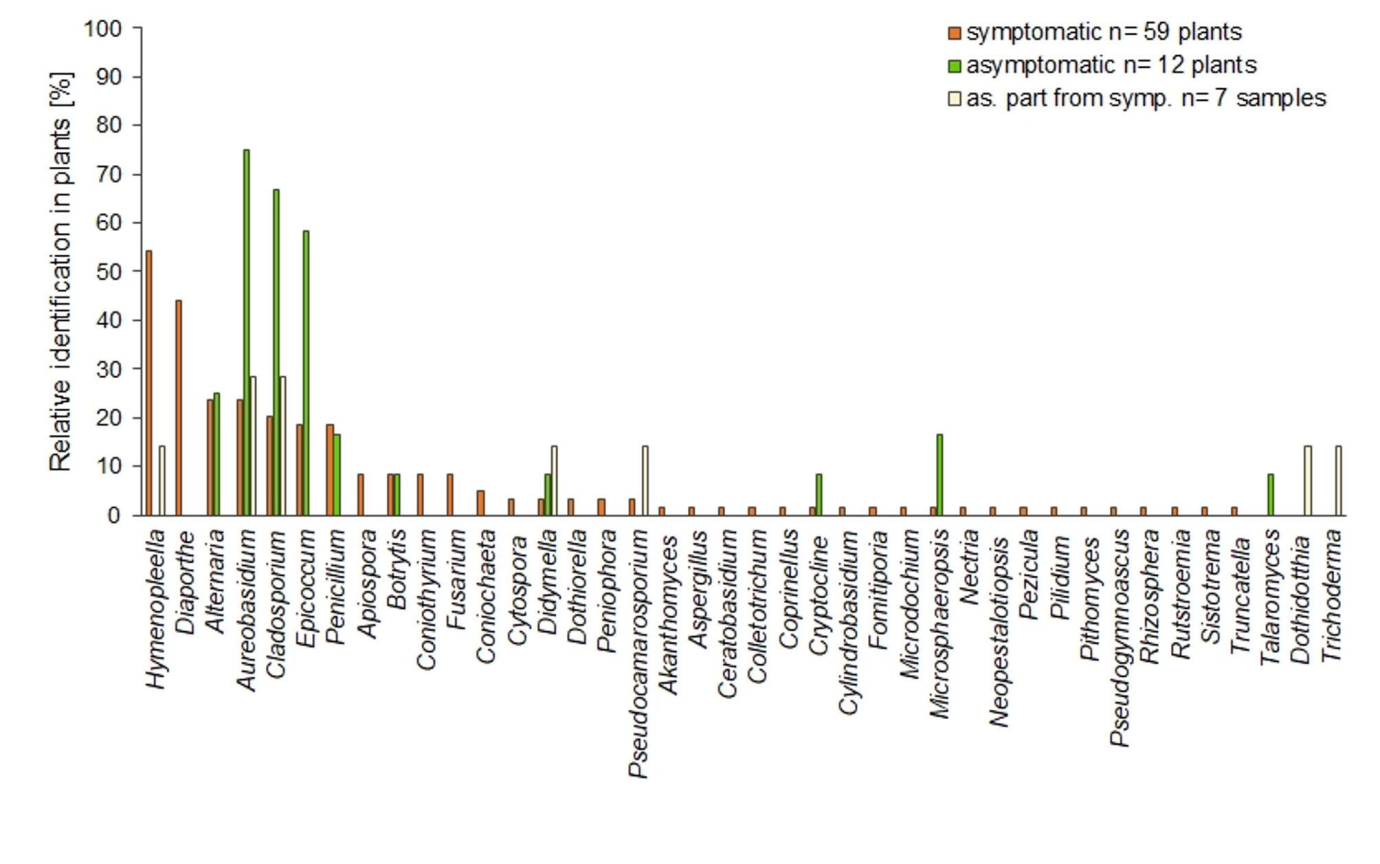
Fungal isolations from shoot samples. Identification of fungal genera obtained from sea buckthorn plants (n counted isolates divided by number of plants sampled). Isolation from 59 symptomatic plants (orange) and 12 asymptomatic plants (green), as well as isolation from asymptomatic parts of symptomatic plants (light yellow, 7 samples). Qualitative identification only considering one genus per plant sampled regardless of total number of obtained isolates of this genus. Identification of isolates by ITS-PCR and Sanger sequencing

Fungal isolation of shoot samples: The isolation rate from symptomatic plants (obtaining any isolate per plant: 85%) was similar to asymptomatic plants (83%). In total, 40 different genera were isolated (Fig. 2). The most frequently isolated fungal genus from symptomatic plants was *Hymenopleella* (55%); it was also detected in two internally asymptomatic specimens sampled from plants with symptomatic sections. All isolates were identified as *Hymenopleella hippophaeicola*. Members of *Diaporthe*, mostly *Diaporthe eres* and to a lesser extent *D. chamaeropsis* and *D. rudis*, were identified in approx. 45% of symptomatic plants but not in asymptomatic plants. *Alternaria* (with approx. 25% each) and *Penicillium* (with approx. 20% each) were detected in both types of plants. Different levels of isolation were obtained for *Aureobasidium* (24% in symptomatic and 75% in asymptomatic), *Cladosporium* (20% and 66%), and *Epicoccum* (18% and 58%). In less than 10% of symptomatic samples, the genera *Apiospora*, *Botrytis*,* Coniothyrium* and *Fusarium* were detected. *Fusarium* isolates were identified as different species, which were *F. acuminatum*, *F. avenaceum*, *F. graminearum*, *F. oxysporum* and *F. sporotrichioides*. *Verticillium*, a potential SBT pathogen, was isolated neither from field nor wild plants during this study.

Amplicon sequencing of shoot samples. The median of combined fungal and Stramenopila reads in SBT shoot samples was 20,861 (min. 1014/max. 71236) for symptomatic and 3335 (min. 1014/max. 71236) for asymptomatic plants (Additional file 1, FO counts). A total of 1237 ASVs were assigned to the kingdom fungi (878 ASVs Ascomycota, 318 ASVs Basidiomycota, 2 ASVs Mucoromycota, 1 ASV each of Chytridiomycota and Olpidiomycota and 37 ASVs Phylum unknown). Two ASVs were assigned to Stramenopila (ASV_35 *Phytophthora aleatoria*, present in three samples, total reads = 14, and ASV_ 5478 *Pythium* sp., present in one sample, total reads = 59). Since only two ASVs could be assigned to the Oomycetes and for better readability, the fungal/Stramenopila communities are referred to as fungal communities. Although some samples had low numbers of ASVs, the rarefaction curves showed a saturation of ASVs (Additional file 4). The most prevalent ASV was assigned to *Hymenopleella* (ASV_3) that was detected in 96% of shoot samples. *Verticillium* was absent in almost all the samples except for four shoot samples where it was detected with low abundance (Additional file 5).

**Fig. 3:**
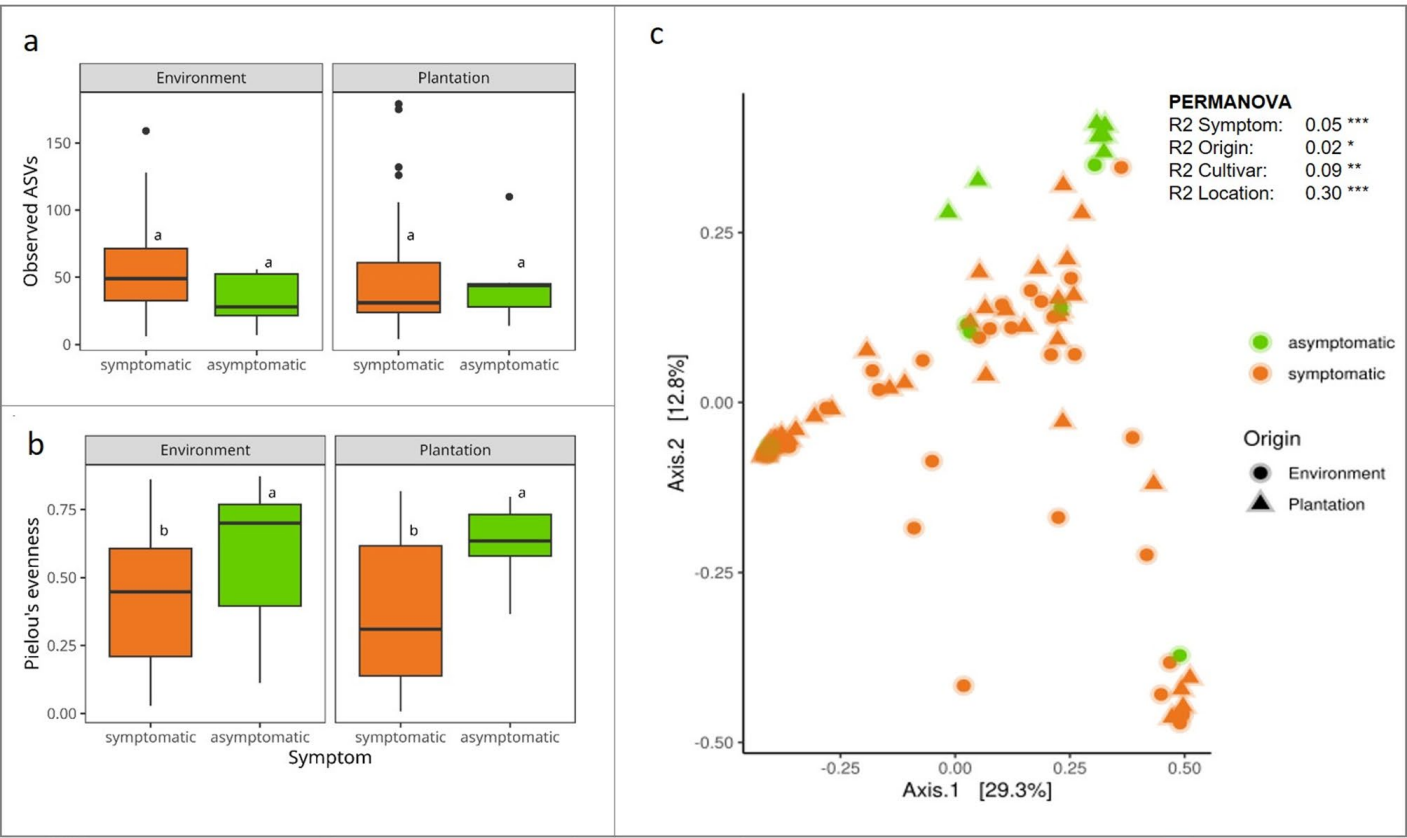
Fungal alpha and beta diversity in sea buckthorn samples: Comparison of **a** observed richness (number of ASVs) and **b** Pielou’s evenness (equally abundance of ASVs). Symptomatic (orange) and asymptomatic plants (green) originating from environment and plantation. ANOVA and a post-hoc Tukey´s HSD correction test with 95% confidence interval. **c** Beta diversity of sea buckthorn shoot samples. Principle coordinates analysis of Bray-Curtis dissimilarities for symptomatic (orange) and asymptomatic plants (green) originating from environment (circle) and plantation (triangle) showing differences in the fungal community of samples. Permutational Multivariate Analyses of Variance Table (PERMANOVA, 10000 permutations) displays variation explained (R2) and significance Pr(> F) (*** ≤ 0.001, **≤ 0.1, *≤ 0.5) for symptom, origin, cultivar, location

Alpha diversity of shoot samples: The alpha diversity displays the fungal community diversity within a sample. Observed richness is the number of ASVs that are detected in the samples. In regard of richness, there were no differences comparing symptomatic and asymptomatic plants across habitats (Fig. 3a). A second measure is Pielou’s evenness referring to the relative abundance of ASVs. ANOVA analysis confirmed only symptom as significant factor to explain changes in the alpha diversity (ANOVA results as F-value and significance level P: Symptom F = 9.165, *P* = 0.00323 (*P* < 0.01**); Origin F = 0.525, *P* = 0.47081 (*P* < 1); Symptom: Origin F = 0.630, *P* = 0.42942 (*P* < 1). Figure 3(b) shows that Pielou’s evenness is significantly reduced in symptomatic samples for each origin (post-hoc Tukey´s test). This implies that for symptomatic plants the dominance of one or more fungal ASVs is increased compared to asymptomatic plants.

Beta diversity of shoot samples: The beta diversity compares fungal communities between samples (Fig. 3c). To assess differences in community composition, Bray-Curtis dissimilarities were calculated and visualized using PCoA. Group separation was statistically tested using PERMANOVA confirming that the different factors studied have a significant effect on the fungal community composition with varying intensity. The main influencing factor was the location (30% of explained variance by PERMANOVA; *P* ≤ 0.001) followed by cultivar (9%; *P* ≤ 0.01), symptom (5%; *P* ≤ 0.001) and origin (environment or plantations) (2%; *P* ≤ 0.05). Despite the high influence of the location on the fungal community composition, the separation of asymptomatic and symptomatic samples is also visualized in the PCoA plot (Fig. 3c). The group separation confirmed by PERMANOVA implies that the symptomatic plants exhibit an altered fungal community composition in comparison to the asymptomatic plants.

**Fig. 4:**
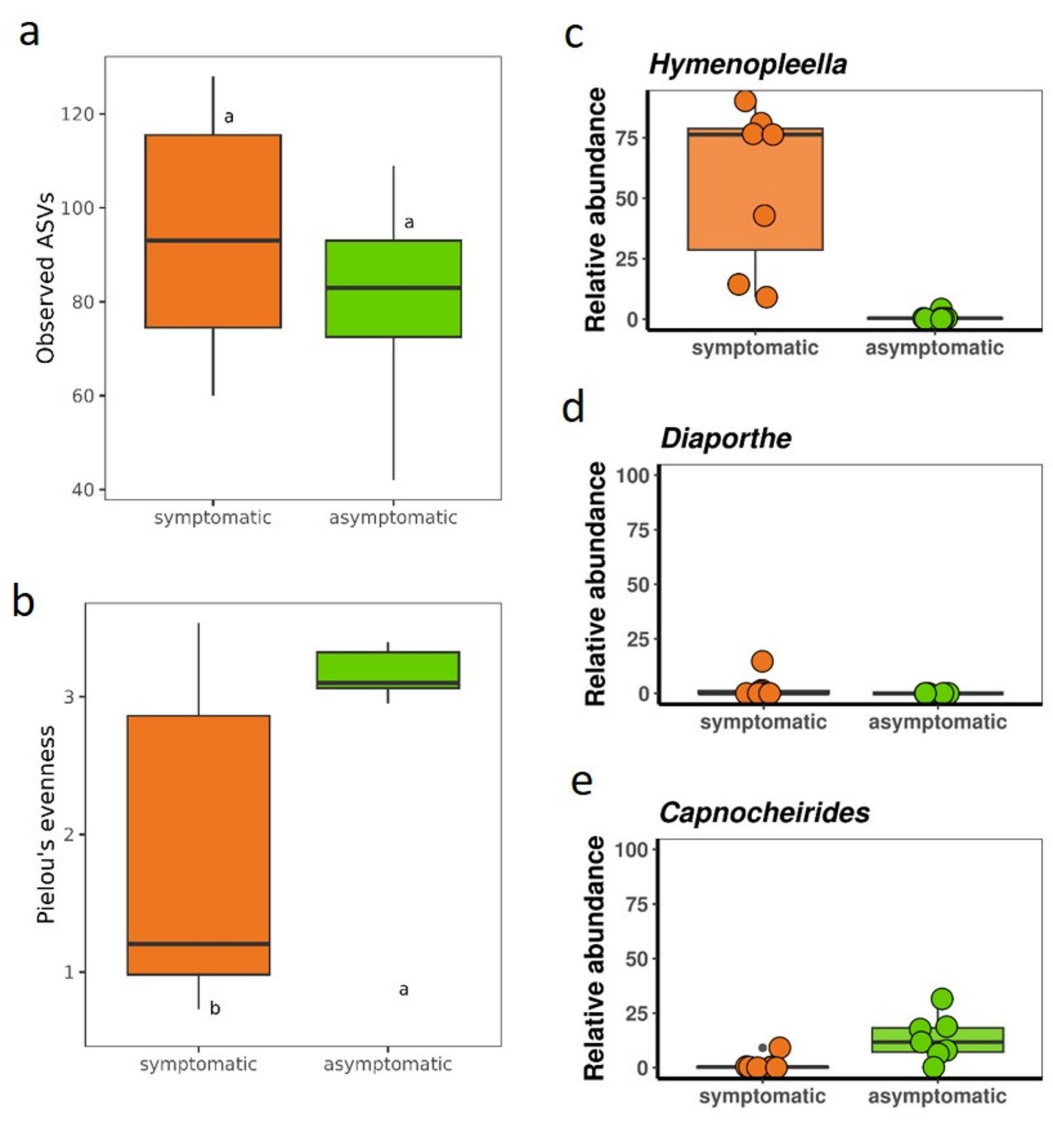
Fungal diversity and abundance in symptomatic (orange) and asymptomatic (green) sections from seven sea buckthorn samples: Comparison of **a** observed richness (number of ASVs) and **b** Pielou’s evenness (equally abundance of ASVs). ANOVA and a post-hoc Tukey´s HSD correction test with 95% confidence interval. **c–e** Relative abundance of the taxa *Hymenopleella*, *Diaporthe* and *Capnocheirides*

Asymptomatic parts from symptomatic plants: As asymptomatic plants were rare, asymptomatic sections from seven plants showing symptoms on other parts were sampled and separately evaluated (Fig. 4). For alpha diversity, Pielou’s evenness was significantly reduced, while no difference could be identified for the number of observed ASVs. In addition, beta diversity showed a clear significant difference in the fungal community with regard to symptoms (R^2^ = 0.41, *p* = 0.0005). *Hymenopleella* had the most negative log_2_ fold change (-6.5) and *Capnocheirides* the highest (4.2). *Hymenopleella* had a mean relative abundance of 0.8% in asymptomatic and 55.8% in symptomatic parts of sampled plants. For *Capnocheirides* the mean relative abundance was higher in asymptomatic sections with 13.5% compared to 1.5% in symptomatic sections. *Diaporthe* was only detectable in one of the symptomatic sections.

**Fig. 5:**
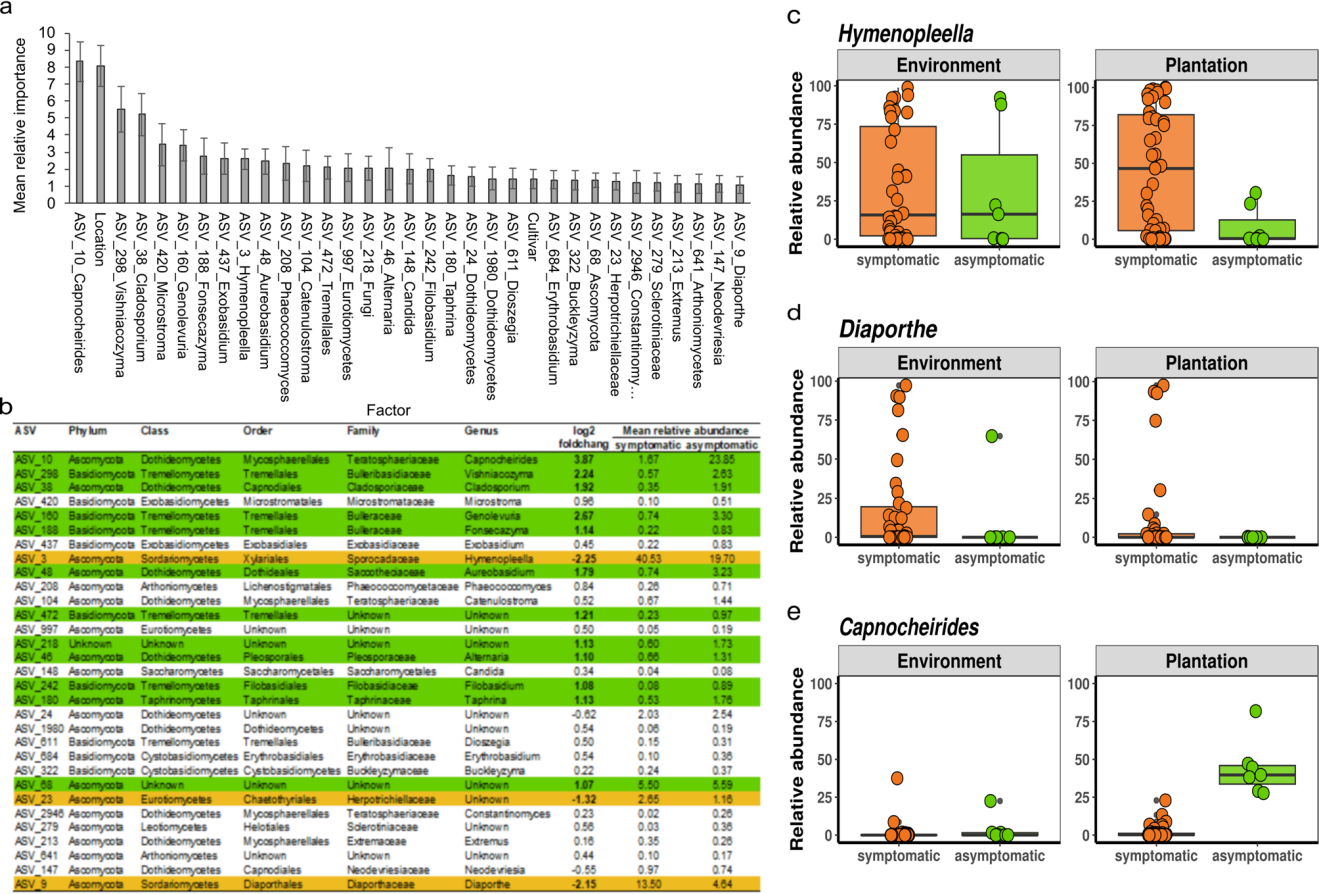
Factors with relative importance in sea buckthorn dieback and relative abundance of fungal taxa.** a** Mean relative importance of top 1% factors in regard of symptoms in SBT dieback context by random forest modelling. Calculation of 100 random forests with standard deviation; **b** Mean relative abundance and phylogeny of ASVs found in top 1% factors of relative importance by random forest modelling, log_2_-fold-change (orange: higher abundance in symptomatic plants and log_2_-fold-change <-1; and green: higher abundance in asymptomatic plants and log_2_-fold-change > 1); **c–e** barplots of relative abundance of single plants for genera *Hymenopleella*, *Diaporthe* and *Capnocheirides*

Random forest modelling: Random forest modelling was done to calculate the mean relative importance (MRI) per variable and to identify those with the highest MRI (≥ 1%) with regard to presence or absence of symptoms (Fig. 5a). The factor with the highest MRI was the ASV *Capnocheirides*, which was only detected in the metabarcoding approach. *Capnocheirides* had a positive log_2_ fold change (3.87) and a higher mean relative abundance in asymptomatic plants (23.8% in asymptomatic and 1.67% in symptomatic plants; Fig. 5b). Especially asymptomatic plants from plantations had a high abundance of *Capnocheirides*, which was less pronounced in environmental plants (Fig. 5e). According to the model, individual location was the second most important factor in terms of symptoms. The MRI of the cultivar is lower than that of the location. The MRI of origin (environment or plantation) was calculated below 1%. Furthermore, there was only a low effect of the origin in the beta diversity and no differences in alpha diversity. Twelve taxa found by the model displayed a positive log_2_ fold change (> 1), while only three taxa had negative values (<-1) which implies that they are more abundant in symptomatic plants. These taxa were (1) *Hymenopleella* (ASV_3), (2) a member of the family *Herpotrichiellaceae* (ASV_23) and (3) *Diaporthe* (ASV_9). *Hymenopleella* was detected in almost every shoot sample, but with high variation in abundance (Fig. 5c). The mean relative abundance at the genus level was 40.5% in symptomatic samples and 19.7% in asymptomatic samples. The genus *Diaporthe* was detected in 45% of the symptomatic plants. Its mean relative abundance was 13.5% in symptomatic and 4.6% in asymptomatic samples (Fig. 5b + d). The member of the family *Herpotrichiellaceae* did not show such high abundances with 2.6% in symptomatic and 1.2% in asymptomatic plants.

**Fig. 6:**
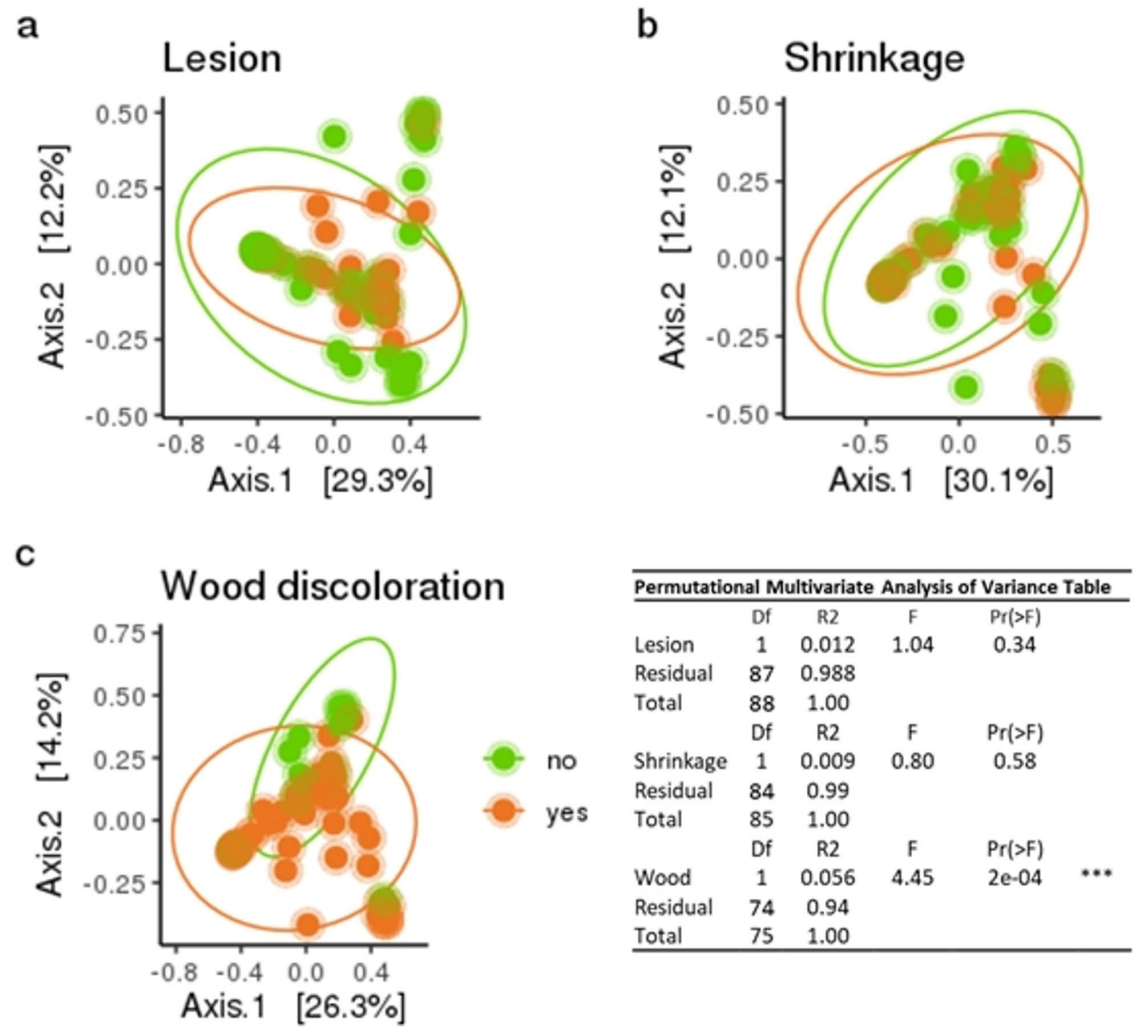
Principle coordinates analysis (PCoA) comparing specific symptoms. Bray-Curtis dissimilarities from log_10_ transformed relative abundance of ASVs. The PCoA comparing the symptoms **a** lesion, **b** shrinkage and **c** wood discolorations. Shoot samples were classified for presence (orange) or absence (green) of the particular symptom. Samples with unclear classification of the respective symptom were removed from the evaluation. Ellipse confidence interval 0.95. Permutational Multivariate Analyses of Variance (PERMANOVA, 10,000 permutations) for each specific symptom separately. Samples showing wood discolorations had a significant different fungal community compared to samples without this symptom

Specific shoot symptoms: In the above described analyses, samples were classified for absence or presence of any symptom. In addition, the presence of lesions, shrinkage and internal wood discoloration was also recorded before starting with the DNA extraction for the amplicon sequencing. The presence or absence for each of the above symptom types was tested as factor of diversity with principle coordinates analysis (Fig. 6). The fungal community was significantly different only for wood discolorations and only samples with wood discolorations showed a significantly reduced Pielou’s evenness (Fig. 6c, Additional file 6). For alpha diversity, the observed number of ASVs was only significantly different for the symptom type lesion in plantation plants (Additional file 6). The mycobiome was mostly affected in sections with symptoms of wood discolorations.

## Discussion

In this study, we investigated for the first time the role of fungi in sea buckthorn (SBT) using a combined approach: fungal communities were in parallel studied by a culture-dependent isolation and a culture-independent metabarcoding approach. Regardless of the approach used, our results have shown that there are significant changes in the fungal communities with regard to dieback symptoms in shoots. These were demonstrated between plants as well as for sections with different symptomatic manifestation on the same plant. Already at the start of our study, both environmental sites and plantations were heavily affected by dieback, and asymptomatic plants were hard to find. Nevertheless, we aimed to investigate the effect of associated fungal communities with regard to the observed dieback symptoms, as it has significant ecological and economic impact. Environmental plants grow in sandy soil in direct proximity to the sea, while plantations are characterized by a higher proportion of humus in the soils, defined plant spacing and harvest pruning every two years. However, similarities in dieback context could be identified for both origins (see alpha and beta diversity). Furthermore, the origin of the plants (plantation vs. environment) was not identified by random forest modelling among the top explanatory variables in dieback context.

*Hymenopleella*, *Diaporthe* (in symptomatic plants) and *Capnocheirides* (asymptomatic plants) were the most relevant genera when comparing symptomless and SBT dieback affected wood mycobiomes from Northern Germany in this study.

***Hymenopleella*** was the most frequently isolated genus from symptomatic plants. Further, ASVs classified as *Hymenopleella* were identified in almost every shoot sample and found to be widespread in German SBT. *Hymenopleella* had the highest mean relative abundance in samples from symptomatic plants and on average its relative abundance was much higher in symptomatic than in asymptomatic samples, and this was evident both between plants and also between symptomatic and asymptomatic parts of the same plant. This finding is supported by our isolation results, as *Hymenopleella* was exclusively isolated from symptomatic plants. This particular association has been described before [30] with *Hymenopleella* occurring on dry SBT branches in the European Alps [73]. provided a detailed description of *H. hippophaeicola* and proposed it as a new name for its generic type *Sphaeria* (= *Lepteutypa) hippophaёs*. *Hymenopleella* has been found elsewhere on sea buckthorn, but the impact on the plants was not exactly evaluated [14]. From our results, we conclude that *Hymenopleella* in low relative abundance is a common endophyte of SBT in Northern Germany, while with high relative abundance it is associated with SBT dieback symptom manifestation.

***Diaporthe*** was also detected in higher abundance in symptomatic shoot samples when compared with asymptomatic samples. In line with this finding, it was exclusively isolated from symptomatic plants. Noteworthy, there was only one sample with relative high *Diaporthe* abundance among the asymptomatic plants (Fig. 4d) and it was absent in other asymptomatic plants. Among the symptomatic plants it was detectable in 46% of the samples and the abundance varied considerably between individual plants. *Diaporthe* together with a complex of other fungal pathogens was described in China and Latvia for SBT plants with symptoms similar to those observed for SBT dieback in Germany [13, 26]. Moreover, *D. eres* and other species have been reported to be pathogenic towards a multitude of woody plants [74–76]. Therefore, we conclude that a high relative abundance of *Diaporthe* is strongly contributing to symptom development of SBT and speculate that *Diaporthe* is one of the causal agents of SBT.

***Capnocheirides*** was detected with higher relative abundance in asymptomatic plants compared to symptomatic plants, especially originating from plantations, but also between asymptomatic and symptomatic sections of the same plant. The genus *Capnocheirides* (order Capnodiales) hitherto is monospecific with only one representative, *Capnocheirides rhododendri* (former classified as *Torula rhododendri)*. It is associated with the formation of sooty mould symptoms on *Rhododendron ferrugineum* lower leaf surface [77, 78]. To our knowledge, *Capnocheirides* was not reported for SBT before. As *C*. *rhododendri* is described as a very slow growing species in vitro this can explain why it was not isolated in our isolation approach during this work. This highlights the synergistic value of combining a culture-dependent approach and molecular-based measures. The current results do not allow a definite conclusion if *Capnocheirides* has a protective function. However, its increased abundance is proposed as a good indicator for the absence of a dysbiosis.

Other taxa of known plant pathogens among the variables with high MRI of the model showed low relative abundances in the symptomatic plants and their pathogenic significance in SBT dieback will be more challenging to assess, especially for those where there were no corresponding isolates such as ASV_23 (no Herpotrichiellaceae taxa among the isolates). Scientific reports from Romania, India and China associated *Verticillium* and *Fusarium* with SBT dieback symptoms [15, 21, 24, 26]. However, these genera were only rarely identified in our study. *Verticillium* was formerly identified in SBT from Germany, but only in healthy looking plants [19]. In Polish dieback affected SBT plants *Alternaria* was the most frequently identified fungal genus, and other potential pathogens such as *Diaporthe* (1.9%) and *F. sporotrichioides* (0.8%) were identified to lesser extents, while *Hymenopleella* or *Verticillium* were not isolated at all [29]. As dieback symptoms might be caused by various fungal pathogens or combinations thereof, specific assessments assigned to particular geographic regions seems necessary. For our study referring exclusively to Northern Germany it is concluded that *Verticillium* and *Fusarium* were not randomly missed due to the chosen approaches, but are either hardly present (*Verticillium*) or are generally present but not specifically associated with dieback symptoms (*Fusarium*). In the present work, only one *Phytophthora* and one *Pythium* ASV were identified in very few plants. As expected, the metabarcoding setup is generally capable of oomycete detection [40], but oomycetes do not seem to be involved in SBT dieback in Northern Germany. It should be kept in mind however, that we cannot exclude the possibility that some ecologically or functionally relevant groups were not detected due to inherent technical and methodological biases. Based on current knowledge and methodological validation, we have no indication that ecologically or pathologically relevant fungal or fungal-like taxa were systematically missed. To further evaluate whether the identified fungal taxa are primary causal agents of SBT dieback, inoculation assays fulfilling Koch’s postulates would be a necessary next step.

Woody plants, e.g. forest and fruit trees, shrubs etc. are commonly affected by a similar range of fungal pathogens, e.g. *Fusarium*, *Diaporthe* or members of latent pathogens within the Botryospheriales [79]. An overall increase of tree mortality was observed for temperate forests in Europe over the last decades and it has been shown that climate change is involved as an additional factor [80]. For Mecklenburg-Western Pomerania, the annual mean temperature increased by 1.3 °C from 1881 to 2018 [81]. In addition, there has also been an increase in warm and dry summers and mild winters in recent years [82]. The relationships between fungal endophytes and plants may change from commensal to pathogenic depending on environmental factors, physiological and developmental state of the plant, and accompanying microorganisms [83]. An imbalance of associated microbiota was often found for woody plants affected by dieback and decline [84] and this was also confirmed in our study demonstrating changes in alpha and beta diversity. One of the grapevine trunk disease agents, *Phaeomoniella chlamydospora*, can be detected in asymptomatic and symptomatic grapevine samples and thereby may constitute an integral part of the grapevine fungal community [85–87]. This might be the same pattern we observed for *Hymenopleella* in this study, which was detected in both symptomatic and asymptomatic SBT shoot samples.

Environmental factors may influence the physiochemical condition of plants, and these changes can eventually result in disease development and distinct symptom expression [88]. By this, potentially deleterious fungi such as *Hymenopleella* or *Diaporthe* might take advantage and dominate the fungal community eventually resulting in symptom development. Our findings derived from both chosen experimental approaches show that *Hymenopleella* is taking advantage of the changes associated with SBT dieback resulting in higher relative abundance and higher frequency of isolation. The subsampling of seven asymptomatic shoots from plants showing symptoms elsewhere revealed that the relative abundance differs within the plant and changes, which suggests that the changes are not systemic and do not affect healthy parts of the plants. Considering the observed relation of the symptom type wood discoloration and a reduced diversity index we recommend evaluation and sampling of these particular symptomatic sections if plantations or wild locations can only be probed with few samples.

Conclusion: It remains unsolved whether changes in the fungal community are the cause or the result of SBT dieback, but the results clearly show that the fungal communities are significantly altered by SBT dieback. *Hymenopleella* dominates the fungal community in dieback affected SBT plants from Northern Germany and *Diaporthe* is suggested as a causal agent.

## Supplementary Information

Below is the link to the electronic supplementary material.

Supplementary Material 1

Supplementary Material 2

Supplementary Material 3

## Data Availability

All data generated or analysed during this study are included in this published article and its supplementary information files. The datasets generated are available: Sanger sequences of representative isolates were deposited in the NCBI gene bank (Accession numbers: PP210697 - PP210858, Additional file 3). Amplicon sequencing raw data was submitted to NCBI database with BioProject and SRA data ID PRJNA1069732 and BioSamples Accession numbers SAMN39620747 - SAMN39621060 (https://www.ncbi.nlm.nih.gov/bioproject/?term=PRJNA1069732).
