# Supplementary material for: Hymenopleella and Diaporthe dominate the fungal community of dieback affected sea Buckthorn from Northern Germany
Source: Environ Microbiome. 2025 Nov 14;20:141. doi: 10.1186/s40793-025-00804-4 (PMC12619318; doi:10.1186/s40793-025-00804-4)
Supplement: Supplementary file 2 — Supplementary Material 2 [file 40793_2025_804_MOESM2_ESM.docx]

Supplementary material

***Hymenopleella* and *Diaporthe* dominate the fungal community of dieback affected sea buckthorn from Northern Germany**

Carolin Popp^1^, Alicia Balbín-Suárez^1^, Falk H. Behrens^2^, Michael Fischer^2^, Wilhelm Jelkmann^1^, and Sabine Kind^1*^

*^1^Julius Kühn Institute - Federal Research Centre for Cultivated Plants, Institute for Plant Protection in Fruit Crops and Viticulture, 69221 Dossenheim, Germany*

*^2^Julius Kühn Institute - Federal Research Centre for Cultivated Plants, Institute for Plant Protection in Fruit Crops and Viticulture, 76833 Siebeldingen, Germany*

*Corresponding author: S. Kind, E-Mail: [Sabine.Kind@julius-kuehn.de](mailto:Sabine.Kind@julius-kuehn.de)

Additional files 1 and 2 are large tables in extra excel files

# Additional file:

## Accession numbers of fungal ITS sequences of representative sea buckthorn isolates deposited in NCBI gene bank

| **Isolate** | **Identification** | **Accession nr.** |
| --- | --- | --- |
| HRS_157-3-A | Neopestalotiopsis sp. | PP210697 |
| HRS_138-1-B | Akanthomyces sp. | PP210698 |
| HRS_138-1-A | Alternaria sp. | PP210699 |
| HRS_139-2-G | Alternaria sp. | PP210700 |
| HRS_18D | Alternaria sp. | PP210701 |
| HRS_20C | Alternaria sp. | PP210702 |
| HRS_23A | Alternaria sp. | PP210703 |
| HRS_27B | Alternaria sp. | PP210704 |
| HRS_167-A | Alternaria sp. | PP210705 |
| HRS_144-2-H | Alternaria sp. | PP210706 |
| HRS_142-2-D | Alternaria sp. | PP210707 |
| HRS_32-1C | Alternaria sp. | PP210708 |
| HRS_169-2-F | Alternaria sp. | PP210709 |
| HRS_171-1-C | Alternaria sp. | PP210710 |
| HRS_122-2-A | Alternaria sp. | PP210711 |
| HRS_171-2-B | Apiospora sp. | PP210712 |
| HRS_120-1-A | Apiospora sp. | PP210713 |
| HRS_139-1-D | Apiospora sp. | PP210714 |
| HRS_152-C | Apiospora sp. | PP210715 |
| HRS_166-C | Apiospora sp. | PP210716 |
| HRS_121-2-D | Apiospora sp. | PP210717 |
| HRS_146-A | Apiospora sp. | PP210718 |
| HRS_35A | Aspergillus sp. | PP210719 |
| HRS_20B | Aureobasidium sp. | PP210720 |
| HRS_8-2A | Aureobasidium sp. | PP210721 |
| HRS_158-1-D | Aureobasidium sp. | PP210722 |
| HRS_156-1-A | Aureobasidium sp. | PP210723 |
| HRS_149-A | Aureobasidium sp. | PP210724 |
| HRS_167-D | Botrytis sp. | PP210725 |
| HRS_166-D | Botrytis sp. | PP210726 |
| HRS_118-1-C | Ceratobasidium sp. | PP210727 |
| HRS_142-2-B | Cladosporium sp. | PP210728 |
| HRS_20A | Cladosporium sp. | PP210729 |
| HRS_24A | Cladosporium sp. | PP210730 |
| HRS_151-D | Cladosporium sp. | PP210731 |
| HRS_144-2-E | Cladosporium sp. | PP210732 |
| HRS_121-2-A | Cladosporium sp. | PP210733 |
| HRS_149-E | Cladosporium sp. | PP210734 |
| HRS_150-E | Cladosporium sp. | PP210735 |
| HRS_119-1-B | Cladosporium sp. | PP210736 |
| HRS_36B | Cladosporium sp. | PP210737 |
| HRS_28A | Colletotrichum sp. | PP210738 |
| HRS_26C | Coniochaeta sp. | PP210739 |
| HRS_14B | Coniochaeta sp. | PP210740 |
| HRS_31-1B | Coniochaeta sp. | PP210741 |
| HRS_102-1-D | Coniothyrium sp. | PP210742 |
| HRS_15C | Coniothyrium sp. | PP210743 |
| HRS_30A | Coniothyrium sp. | PP210744 |
| HRS_19C | Coniothyrium sp. | PP210745 |
| HRS_16D | Coniothyrium sp. | PP210746 |
| HRS_26E | Coniothyrium sp. | PP210747 |
| HRS_122-3-D | Cryptocline sp. | PP210748 |
| HRS_132-1-B | Cryptocline sp. | PP210749 |
| HRS_172-D | Cylindrobasidium sp. | PP210750 |
| HRS_137-1-F | Cytospora sp. | PP210751 |
| HRS_155-D | Cytospora sp. | PP210752 |
| HRS_172-E | Diaporthe sp. | PP210753 |
| HRS_137-2-B | Diaporthe sp. | PP210754 |
| HRS_156-1-B | Diaporthe sp. | PP210755 |
| HRS_167-C | Diaporthe sp. | PP210756 |
| HRS_24C | Diaporthe sp. | PP210757 |
| HRS_23E | Diaporthe sp. | PP210758 |
| HRS_25D | Diaporthe sp. | PP210759 |
| HRS_158-1-G | Diaporthe sp. | PP210760 |
| HRS_118-2-B | Diaporthe sp. | PP210761 |
| HRS_102-A | Diaporthe sp. | PP210762 |
| HRS_8-1A | Diaporthe sp. | PP210763 |
| HRS_18B | Diaporthe sp. | PP210764 |
| HRS_22A | Diaporthe sp. | PP210765 |
| HRS_27E | Diaporthe sp. | PP210766 |
| HRS_19B | Diaporthe sp. | PP210767 |
| HRS_161-2-C | Diaporthe sp. | PP210768 |
| HRS_139-2-A | Didymella sp. | PP210769 |
| HRS_154-C | Didymella sp. | PP210770 |
| HRS_118-1-B | Dothiorella sp. | PP210771 |
| HRS_26B | Dothiorella sp. | PP210772 |
| HRS_140-B | Didymella sp. | PP210773 |
| HRS_153-A | Dothidotthia sp. | PP210774 |
| HRS_153-C | Didymella sp. | PP210775 |
| HRS_161-1-A | Epicoccum sp. | PP210776 |
| HRS_102-1-C | Epicoccum sp. | PP210777 |
| HRS_139-2-C | Epicoccum sp. | PP210778 |
| HRS_157-2-D | Epicoccum sp. | PP210779 |
| HRS_168-C | Epicoccum sp. | PP210780 |
| HRS_172-A | Epicoccum sp. | PP210781 |
| HRS_121-3-A | Epicoccum sp. | PP210782 |
| HRS_147-B | Epicoccum sp. | PP210783 |
| HRS_142-2-G | Epicoccum sp. | PP210784 |
| HRS_143-2-C | Epicoccum sp. | PP210785 |
| HRS_144-2-A | Epicoccum sp. | PP210786 |
| HRS_23B | Epicoccum sp. | PP210787 |
| HRS_32-2A | Epicoccum sp. | PP210788 |
| HRS_33A | Epicoccum sp. | PP210789 |
| HRS_145-A | Epicoccum sp. | PP210790 |
| HRS_140-D | Epicoccum sp. | PP210791 |
| HRS_119-1-C | Epicoccum sp. | PP210792 |
| HRS_138-2-B | Fomitiporia hippophaeicola | PP210794 |
| HRS_158-2-A | Fusarium sp. | PP210795 |
| HRS_160-2-F | Fusarium sp. | PP210796 |
| HRS_170-B | Fusarium sp. | PP210797 |
| HRS_25-A | Hymenopleella sp. | PP210798 |
| HRS_1-2-A | Hymenopleella sp. | PP210799 |
| HRS_30C | Hymenopleella sp. | PP210800 |
| HRS_32-1B | Hymenopleella sp. | PP210801 |
| HRS_31-1A | Hymenopleella sp. | PP210802 |
| HRS_6-1D | Hymenopleella sp. | PP210803 |
| HRS_7-1A | Hymenopleella sp. | PP210804 |
| HRS_12D | Hymenopleella sp. | PP210805 |
| HRS_27F | Hymenopleella sp. | PP210806 |
| HRS_2-2C | Hymenopleella sp. | PP210807 |
| HRS_13C | Hymenopleella sp. | PP210808 |
| HRS_16B | Hymenopleella sp. | PP210809 |
| HRS_19A | Hymenopleella sp. | PP210810 |
| HRS_28D | Hymenopleella sp. | PP210811 |
| HRS_139-2-B | Hymenopleella sp. | PP210812 |
| HRS_138-2-A | Hymenopleella sp. | PP210813 |
| HRS_152-D | Hymenopleella sp. | PP210814 |
| HRS_157-2-A | Hymenopleella sp. | PP210815 |
| HRS_163-1-B | Hymenopleella sp. | PP210816 |
| HRS_161-1-D | Hymenopleella sp. | PP210817 |
| HRS_102-B | Hymenopleella sp. | PP210818 |
| HRS_171-1-B | Hymenopleella sp. | PP210819 |
| HRS_170-F | Hymenopleella sp. | PP210820 |
| HRS_161-2-D | Alternaria sp. | PP210821 |
| HRS_102-2-D | Microdochium sp. | PP210822 |
| HRS_144-2-G | Microsphaeropsis sp. | PP210823 |
| HRS_154-B | Nectria sp. | PP210825 |
| HRS_122-1-A | Penicillium sp. | PP210827 |
| HRS_153-D | Coniothyrium sp. | PP210828 |
| HRS_4-2-A | Coniothyrium sp. | PP210829 |
| HRS_6-1-E | Penicillium sp. | PP210830 |
| HRS_11A | Penicillium sp. | PP210831 |
| HRS_118-3-A | Penicillium sp. | PP210832 |
| HRS_121-1-B | Penicillium sp. | PP210833 |
| HRS_156-3-C | Penicillium sp. | PP210834 |
| HRS_157-3-F | Penicillium sp. | PP210835 |
| HRS_160-1-B | Penicillium sp. | PP210836 |
| HRS_163-1-A | Penicillium sp. | PP210837 |
| HRS_144-2-B | Penicillium sp. | PP210838 |
| HRS_16A | Peniophora sp. | PP210839 |
| HRS_17A | Peniophora sp. | PP210840 |
| HRS_17B | Pezicula sp. | PP210841 |
| HRS_18E | Microsphaeropsis sp. | PP210844 |
| HRS_156-3-F | Pilidium sp. | PP210845 |
| HRS_139-2-D | Pithomyces sp. | PP210846 |
| HRS_156-2-C | Pseudocamarosporium sp. | PP210847 |
| HRS_118-3-E | Pseudocamarosporium sp. | PP210848 |
| HRS_155-B | Pseudocamarosporium sp. | PP210849 |
| HRS_12E | Pseudogymnoascus sp. | PP210850 |
| HRS_9-1A | Rhizosphaera sp. | PP210851 |
| HRS_163-2-C | Rutstroemia sp. | PP210852 |
| HRS_160-1-C | Sistotrema sp. | PP210853 |
| HRS_18C | Rhizosphaera sp. | PP210854 |
| HRS_21A | Talaromyces sp. | PP210855 |
| HRS_156-2-A | Trichoderma sp. | PP210856 |
| HRS_152-F | Truncatella sp. | PP210857 |
| HRS_154-A | Truncatella sp. | PP210858 |


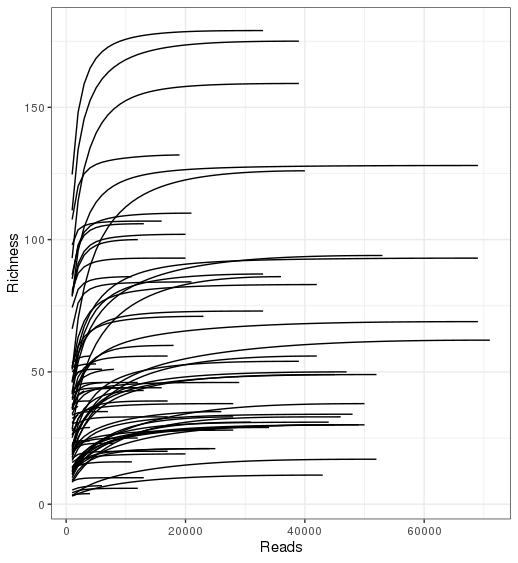


# Additional file:

## **Rarefaction curves from shoot material of SBT plants**. Although some samples had only a few reads, curves show a saturation. Evaluation of amplicon sequence data was carried out for samples ≥ 1000 fungal and oomycetes reads

# Additional file:

## **Abundance of Verticillium** in SBT sequencing data of shoot samples and the mock community. Number of total fungal and oomycetes (FO) reads, total number of all *Verticillium* ASVs, and relative abundance of *Verticillium*

| **Material** | **Shoot** | | | | **Mock** |
| --- | --- | --- | --- | --- | --- |
| Sample | 012 | 041 | 093 | 143 | 309 |
| Total FO reads | 44905 | 33037 | 45802 | 35874 | 84273 |
| Total *Verticillium* reads | 12 | 1234 | 8 | 161 | 34261 |
| **Rel. abundance *Verticillium* %** | **0.03** | **3.74** | **0.02** | **0.45** | **40.65** |


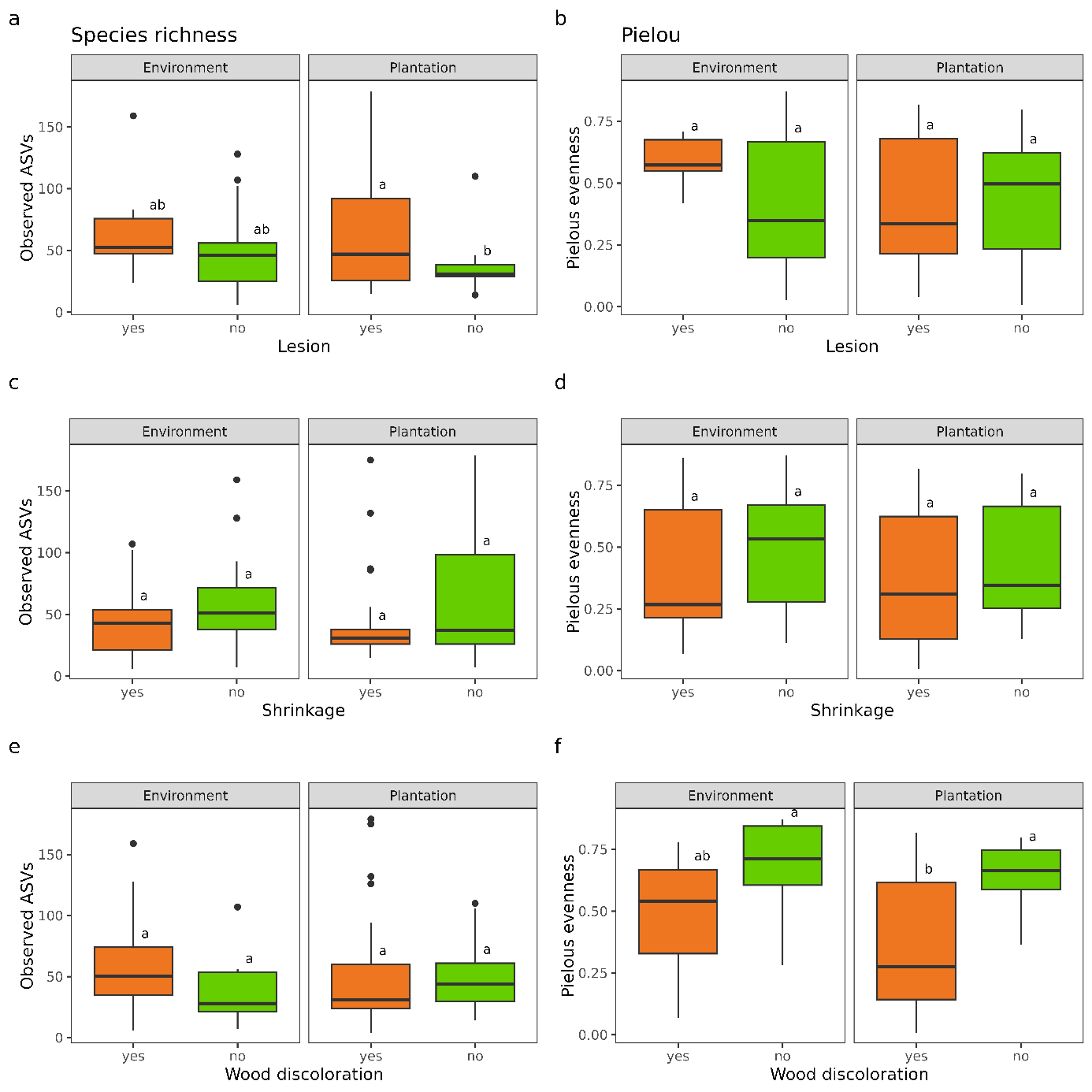


# Additional file:

## **Alpha diversity for specific symptoms shoot samples.** Comparison of observed richness (left side) and Pielou’s evenness (right side) for shoot symptoms a,b) Lesion, c,d) shrinkage and e,f) wood discolorations. Symptom was present (yes, orange) or absent (no, green). Only for symptom wood discoloration a significant reduction of Pielou’s evenness could be observed. ANOVA and Tukey post hoc test 95% confidence interval
